# Clinical Decision-Making Case: Seizing the Diagnosis: Eclampsia

**DOI:** 10.21980/J8.52338

**Published:** 2025-12-31

**Authors:** Samuel Parnell, Joshua Ginsburg, Brian Milman, Marshall Howell

**Affiliations:** * UT Southwestern Medical Center, Department of Emergency Medicine, Dallas, TX

## Abstract

**Audience:**

This practice certifying exam case is intended for emergency medicine residents.

**Introduction:**

The American Board of Emergency Medicine (ABEM) certification process is currently undergoing a significant transformation, with the new ABEM Certifying Exam replacing the long-standing ABEM Oral Exam, which has been in place since 1980.^1^ The Certifying Exam will utilize a new exam format to evaluate different competencies compared to the Oral Exam, including high-stakes communication, managing difficult conversations, patient-centered communication, clinical decision-making, team management, leadership, procedural skills, ultrasound skills, reassessment, troubleshooting, task switching, and prioritization.^1^ This shift has understandably generated a degree of apprehension among Emergency Medicine (EM) residents preparing for their board exams. The new exam emphasizes several new cases and scenarios, including Clinical Decision-Making (CDM) cases. These structured discussions require residents to articulate their thought processes and justify their clinical choices while navigating simulated Emergency Department (ED) encounters with undifferentiated patients.^1^

Although the CDM cases are similar in length (15 minutes) and format to the Structured Interview cases from the prior Oral Exam (structured discussion eliciting the steps necessary to diagnose and treat a patient with an undifferentiated presentation), ABEM has established new scoring criteria and objectives for the CDM cases.^2^ Therefore, to effectively prepare residents for this evolving assessment landscape, relevant and challenging example CDM case scenarios are essential. This CDM case focuses on eclampsia, defined as seizures occurring in a woman with pre-eclampsia or gestational hypertension during the peripartum period. This condition is a relatively rare, yet critical, emergency at the intersection of obstetrics and neurology that carries significant maternal and fetal risk and demands prompt recognition and decisive management. This case aligns directly with the exam’s emphasis on high-acuity, low-occurrence presentations and provides a realistic and challenging opportunity to hone crucial clinical decision-making skills. It ensures that residents are not only well-prepared for the high-stakes Certifying Exam but are also capable of effectively managing this critical obstetric emergency in actual clinical practice.

**Educational Objectives:**

By the end of this Mock Certifying Exam session, learners should be able to: 1) demonstrate familiarity with the Clinical Decision-Making case format and structure, 2) elicit relevant historical information and connect that information to the diagnosis of eclampsia, 3) describe and interpret physical exam findings and their significance in establishing a pertinent differential diagnosis, which includes eclampsia, 4) initiate appropriate diagnostic testing, interpret results accurately, and formulate a stabilization and treatment plan for a patient with eclamptic seizures, and 5) reassess the patient’s condition, modify the management plan as needed, provide relevant anticipatory guidance for disposition, and articulate the clinical decision-making rationale at each stage of the encounter.

**Educational Methods:**

This educational intervention employed a simulated ABEM Certifying Examination case. The initial case, focusing on the critical presentation of eclampsia, was developed collaboratively by a team of Emergency Medicine education faculty at a large, urban, three-year EM residency program. The structure and format were modeled after examples of the new CDM cases provided on the ABEM website. The CDM format involves a dynamic patient encounter in which the learner progresses through different phases of care, making diagnostic and therapeutic decisions and justifying their rationale to an examiner.^1^

A pilot phase was conducted to ensure realism, educational value, and appropriate timing for the examination format. Four recent graduates of the EM residency program participated in this pilot: two fellows (in Emergency Medical Services and Medical Education, respectively) and two actively practicing attending physicians in community Emergency Department settings. These individuals engaged with the case as if they were taking the actual Certifying Examination, providing detailed feedback on the clarity of the scenario, the appropriateness of the clinical challenges presented, and the overall flow and timing of the case. Based on the insights gained from pilot testing, the case underwent refinement and adjustments to optimize its effectiveness as an assessment and learning tool.

The finalized eclampsia CDM case was then implemented in a testing phase involving eighteen PGY-3 (Post Graduate Year-3) EM residents. These residents participated in a simulated certifying examination experience, with two faculty members acting as examiners for each resident physician in individual exam rooms, closely resembling the actual testing environment.^1^ The examiners sat across a table from the examinee, and a separate screen was utilized to display the case stimuli and task sheet during the case. The case sessions were limited to a maximum of 15 minutes. While this CDM case was incorporated into a Mock Certifying Exam session with multiple other cases and scenarios, it could also be employed in isolation using a virtual or in-person format. Following the Mock Certifying Exam, a group debrief was conducted in-person. This session provided collective feedback to resident examinees and faculty examiners regarding the eclampsia case, the CDM case format, and key clinical learning points.

**Research Methods:**

Following each simulated case encounter, faculty examiners evaluated resident performance using a standardized scoresheet. The scoresheet, derived from ABEM Certifying Exam Score Guidelines, assessed nine domains: focused history, physical exam interpretation, differential diagnosis, diagnostics, patient stabilization, reassessment, disposition, clinical reasoning, and pathophysiology.^2^

These nine domains were collated into the standard JETem scoring rubric, which includes 25 points spread among categories associated with the CDM format. In addition to the faculty assessment, residents anonymously completed a two-question post-case evaluation. This evaluation employed a 5-point Likert scale (1 = poor, 5 = excellent) to assess the overall quality of the case, characterized by how well the session met educational objectives and resident expectations, and the degree to which the case enhanced understanding of the certifying exam format (1 = strongly disagree, 5 = strongly agree).

**Results:**

In total, 18 residents completed the case, and 17/18 residents (94.4%) completed the post-case evaluation. All residents identified eclampsia as one of the top three relevant differential diagnoses, administered intravenous magnesium as part of the treatment, and appropriately admitted the patient. However, antihypertensive medications were the most overlooked treatment modality.

The mean quality rating of the case was 4.94/5, and 100% of resident respondents rated the overall quality of the case as “very good” or “excellent” on a 5-point Likert scale. Furthermore, 100% of respondents agreed or strongly agreed that the case enhanced their understanding of the certifying exam format.

**Discussion:**

The implementation of this CDM case proved highly effective in preparing EM residents for the complexities of clinical practice and the new challenge of understanding the Certifying Exam CDM format. The case was exceptionally well received, with a mean quality rating of 4.94 out of 5, and 100% of resident respondents rating its overall quality as “very good” or “excellent.” Furthermore, 100% of examinees agreed or strongly agreed that the case significantly enhanced their understanding of the certifying exam format, underscoring its utility as a preparatory tool for the newly formatted ABEM Certifying Exam.

From its implementation, we learned that high-fidelity mock cases, particularly those involving critical scenarios like eclampsia, are invaluable for assessing and refining clinical decision-making skills. Eclampsia is a high-yield case due to its critical nature, requiring a wide differential diagnosis for seizures, a deep understanding of pathophysiology, and the rapid initiation of diagnostic workup and treatment to mitigate significant morbidity and mortality. This type of simulation-based training provides a safe but realistic testing environment for residents to gain experience with rare or high-acuity situations they may not frequently encounter in clinical practice.

The main takeaway from this experience is the critical importance of proactive, targeted preparation for the evolving ABEM Certifying Exam. The overwhelmingly positive resident feedback and performance scores demonstrate that such simulated experiences are highly effective in enhancing understanding of the exam format. By providing a realistic, low-stakes environment for decision-making and targeted feedback, these mock cases directly contribute to residents’ preparedness for board certification and, more importantly, for safe, high-quality independent practice in emergency medicine.

**Topics:**

Clinical decision-making case, eclampsia, seizure, post-partum, certifying exam, ABEM.

## USER GUIDE

**Table:** 

List of Resources:	
Abstract	186
User Guide	189
For Examiner Only	192
Certifying Exam Assessment	200
Stimulus	202
Debriefing and Evaluation Pearls	210

**Learner Audience:**

Junior Residents, Senior Residents

**Time Required for Implementation:**

Case: Clinical Decision-Making cases are 15 minutes as directed by American Board of Emergency Medicine (ABEM).

Debriefing: 10 minutes

**Recommended number of learners per instructor:**

1 learner paired with two instructors

**Topics:**

Clinical decision-making case, eclampsia, seizure, postpartum, certifying exam, ABEM.

**Objectives:**

By the end of this structured interview case, learners will be able to:

1. Demonstrate familiarity with the Clinical Decision-Making (CDM) case format and structure.
2. Elicit relevant historical information and connect that information to the diagnosis of eclampsia.
3. Describe and interpret physical exam findings and their significance in establishing a pertinent differential diagnosis, which includes eclampsia.
4. Initiate appropriate diagnostic testing, interpret results accurately, and formulate a stabilization and treatment plan for a patient with eclamptic seizures.
5. Reassess the patient's condition, modify the management plan as needed, provide relevant anticipatory guidance for disposition, and articulate the clinical decision-making rationale at each stage

### Linked objectives, methods and results

The design of this mock Certifying Examination case, structured as a CDM interview, was developed to align with the specific educational objectives and scoring criteria for the ABEM Certifying Exam. The CDM format directly facilitates the achievement of our objectives by providing a dynamic, interactive environment that mirrors the cognitive processes inherent in Emergency Medicine practice.^1^ This format allows examiners to observe and evaluate a resident’s thought process in real-time, assessing not just what they know but how they apply that knowledge under time constraints.

The selection of the CDM format was deliberate, rooted in its ability to assess complex clinical judgment and its direct relevance to the new ABEM Certifying Exam. While the prior ABEM Oral Board exam included structured interview cases that similarly assessed clinical judgment and reasoning through stages of patient care, the new Certifying Exam’s “Clinical Decision-Making” cases are explicitly discussion-based, focusing on prioritization and interaction with standardized clinical information. This mock exam directly simulates these CDM cases, allowing residents to practice and become familiar with the new format and scoring requirements they will encounter. Our content development was guided by a conceptual framework of clinical judgment, which outlines essential cognitive steps: recognizing cues, analyzing information, defining a hypothesis, generating solutions, taking action, and evaluating outcomes. This framework underpins each of the stated objectives. For instance, the eclampsia case was chosen as a high-yield scenario because it necessitates rapid application of this entire framework. Managing eclampsia requires residents to quickly recognize critical cues (eg, new-onset seizures in a postpartum woman with elevated blood pressure), analyze complex information (eg, differential diagnosis for seizures and pathophysiology of preeclampsia), generate and implement solutions (eg, magnesium sulfate and blood pressure control), and continuously reassess the patient’s response to treatment modalities. This comprehensive demand for integrated clinical decision-making makes eclampsia an ideal case to assess all the specified objectives within this conceptual framework, preparing residents for the high-stakes, real-world scenarios they will face in practice and on the certifying exam.

### Recommended pre-reading for instructor

Instructors should review the example case summaries, scoring information, and sample case videos related to the ABEM Certifying Exam and specifically the Clinical Decision-Making case on the ABEM website: https://www.abem.org/get-certified/certifying-exam/certifying-exam-content.^1^–^2^ These resources will help ensure that faculty instructors are familiar with the content, format, timing, and scoring of the Clinical Decision-Making case. For additional information about the diagnosis and treatment of eclampsia, instructors can review Chapter 100 in Tintinalli’s *Emergency Medicine: A Comprehensive Study Guide*, or Chapter 177 in *Rosen’s Emergency Medicine: Concepts and Clinical Practice*.^3^–^4^

### Results and tips for successful implementation

The eclampsia case was collaboratively developed by a team of experienced EM faculty at a large, urban, three-year EM residency program. To ensure authenticity, educational relevance, and appropriate pacing, the case underwent a pilot phase involving four recent residency graduates: two fellows (Emergency Medical Services and Medical Education) and two practicing community EM attendings who were all actively preparing for their ABEM board exams. Participants completed the case under exam-like conditions and provided structured feedback on case clarity, clinical content, and timing.

Based on this feedback, the case was refined to enhance its utility as both an assessment and educational tool. The final version was administered to 18 PGY-3 residents during a mock certifying exam. Each resident was evaluated by two faculty examiners in an exam room using a standardized scoresheet designed to assess clinical decision-making, diagnostic reasoning, and management within the eclampsia scenario. The scoresheet was adapted from ABEM guidelines to best align with board expectations. During the case, one examiner primarily facilitated the case dialogue, while the other primarily recorded responses, managed scoring, and monitored time. Cases were limited to 15 minutes, followed by a 5-minute examiner discussion to collaboratively finalize the resident’s score.

All 18 residents completed the case, with 17 (94.4%) submitting anonymous post-case evaluations. The case was highly rated, with a mean quality score of 4.94 out of 5. All respondents (100%) rated the overall quality as “very good” or “excellent.” Additionally, 100% agreed or strongly agreed that the case enhanced their understanding of the ABEM Certifying Examination format, highlighting its value as a preparatory tool for the new exam. Every resident completed the case within the 15 allotted minutes, and all residents identified eclampsia as the correct final diagnosis. Furthermore, every resident administered intravenous magnesium as part of the management plan and appropriately admitted the patient to the hospital. However, antihypertensive medications were the most overlooked treatment modality.

Following case completion, a group debrief was conducted with participating residents and faculty examiners. Overall feedback was highly positive, with residents noting that the case and format felt realistic and manageable within the 15-minute time frame. Several residents suggested that additional prompts highlighting the patient’s persistent hypertension, such as repeat vital signs, would reinforce the need for antihypertensive management. The faculty also recommended revising the case script to include updated vitals during the treatment phase and incorporating examiner prompts to explore additional treatment options if residents only administered intravenous magnesium and/or benzodiazepines. These changes were subsequently made, and a prompt with repeat vitals was incorporated into the final case included in this manuscript.

This postpartum eclampsia CDM case provides a valuable opportunity to prepare Emergency Physicians for their Certifying Exam and clinical practice. Engagement with this high-fidelity, clinically pertinent scenario is expected to enhance residents’ clinical decision-making capabilities, thereby fostering preparedness for the Certifying Examination and competent, timely management of this life-threatening obstetric emergency in clinical practice.

**Table:** Clinical Decision-Making Case

Resident #	Format Question	Quality Question
**1**	5	5
**2**	5	5
**3**	4	5
**4**	5	5
**5**	5	5
**6**	5	5
**7**	5	5
**8**	5	5
**9**	5	5
**10**	5	5
**11**	5	5
**12**	4	4
**13**	5	5
**14**	5	5
**15**	5	5
**16**	5	5
**17**	5	5
**N**	17	17
**Response Rate**	94.44%	94.44%
**Average**	4.8823529	4.9411765

**Format Question:** This case increased my understanding of the certifying exam format. [strongly disagree - 1, disagree - 2, neutral - 3, agree - 4, strongly agree - 5]

**Quality Question:** How would you rate the overall quality of this case? [poor - 1, fair - 2, good - 3, very good - 4, excellent - 5]

## Figures and Tables

**Table:** 

**I. History**		**Yes**	**No**
**1**	*Asks about history of seizures?*		
**2**	*Asks about recent head trauma, pregnancy, or illness?*		
**3**	*Appropriate Rationale for PROMPT 2 #1?*		
**4**	*Appropriate Rationale for PROMPT 2 #2?*		
**II. Physical Examination**			
**5**	*Assesses for signs of trauma*		
**6**	*Examines for lower extremity edema*		
**7**	*Performs a complete neurological exam (orientation, strength, sensation, coordination, cranial nerves, reflexes, etc.)*		
**8**	*Appropriate Rationale for PROMPT 4?*		
**III. Differential Diagnosis**			
**9**	*Eclampsia listed on differential*		
**10**	*Second appropriate differential diagnosis listed from scoring guidelines (SAH, ICH, PRES, hypertensive emergency, thyroid storm, intracranial mass, intoxication, EtOH/drug withdrawal, meningitis/encephalitis, epilepsy, hypoglycemia, hyponatremia, syncope)*		
**11**	*Third appropriate differential diagnosis listed from scoring guidelines (SAH, ICH, PRES, hypertensive emergency, thyroid storm, intracranial mass, intoxication, EtOH/drug withdrawal, meningitis/encephalitis, epilepsy, hyponatremia, hypoglycemia, syncope)*		
**IV. Diagnostic Studies**			
**12**	*Orders pertinent lab work including POC Glucose*		
**13**	*Orders CT Head*		
**14**	*Appropriate rationale for PROMPT 7 #1?*		
**15**	*Correctly interprets labs identifying proteinuria*		
**16**	*Correctly interprets CT Head*		
**V. Treatment and Other Actions**			
**17**	*Orders IV Magnesium Sulfate with correct dosing (4–6g IV)*		
**18**	*Orders IV anti-hypertensives with correct dosing (IV labetalol 10–40 mg, IV hydralazine 10–20 mg, or IV nicardipine infusion 5 mg/hr – 15mg/hr)*		
**19**	*Appropriate rationale for PROMPT 10 #1?*		
**20**	*Appropriate rationale for PROMPT 10 #2?*		
**VI. Final Diagnosis**			
**21**	*Names eclampsia as the final diagnosis*		
**VII. Broad Principles**			
**22**	*Correctly discusses immediate management of status epilepticus including supportive care and/or benzodiazepine treatment*		
**VIII. Disposition**			
**23**	*Admits the patient to an appropriate service - OB/GYN or ICU with OB/GYN consulted*		
**24**	*Appropriate rationale for PROMPT 13?*		
**IX. Transitions of Care**			
**25**	*Effectively discusses the diagnosis and disposition with the patient and her husband*		

Suggested passing cutoff for this case is 70% or ≥18/25 points.

**Table:** 

**Candidate Task Sheet**	
**#1**	**Patient Information Form**
**#2**	**Additional History**
**#3**	**Physical Exam Findings**
**#4**	**Electrocardiogram**
**#5**	**Laboratory Results**
**#6**	**Computed Tomography of the Head**

**Table:** Clinical Decision-Making Task Sheet

**CASE PARAMETERS**
This is a 15-minute case You will interact with two examiners. This is an interview style without role playing; you should simply reply to the questions asked. You may be interrupted to move you through the case; this is not a reflection of your performance.
**PATIENT INFORMATION**
42-year-old female presenting by EMS with seizure-like activity.
**VITAL SIGNS**
BP: 195/100 mmHg P: 120 bpm R: 16 pm Oxygenation: 95% on room air Temp: 37.5º C Weight: 80 kg
**TASK STATEMENT**
Your tasks are as follows: List pertinent elements of a focused history and physical exam Develop an appropriate differential and/or provisional diagnosis Select and interpret appropriate studies Articulate appropriate patient management Determine the appropriate disposition for the patient Update the patient and family on the disposition and plan of care

**STIMULUS 1:** Emergency Department Patient Information Form

**Patient Information**	
**Patient Name**	Jade Ramirez
**Age**	42 years old
**Gender**	Female
**Method of Arrival**	EMS
**Presenting Complaint**	Seizure-like activity
**Person Providing History**	EMS and Husband
**General Appearance**	Sleepy and confused
**Initial History of Present Illness**	The patient presents by EMS after an episode of seizure-like activity at home. Just prior to arrival, the patient had an episode where she was unresponsive and had shaking of the arms and legs. She has been sleepy and confused ever since that episode.
**Vital Signs on Initial Presentation**	BP: 195/100 mmHg P: 120 bpm R: 16 pm Oxygenation: 95% on room air Temp: 37.5º C Weight: 80 kg

**STIMULUS 2:** Additional History

**History of Present Illness/Description of Event**	
*Source: The patient is confused and not answering questions. History is provided by husband and EMS.* Jade is a 42-year-old female who was brought in for seizure-like activity. She is seven days postpartum from an uncomplicated spontaneous vaginal delivery. The patient had standard prenatal care with negative Group B Strep screen and mildly elevated blood pressure during the pregnancy that was monitored. She has been complaining of a mild headache and blurry vision for the past three days. Today, the patient had an episode of “jerking her arms and legs” for about two to three minutes while sitting at the table after eating breakfast. The husband states “her eyes rolled back, and she was jerking and not responding during that time.” He also reports she bit her tongue and urinated on herself. She was seated during the episode and did not fall or hit her head. She has been confused and sleepy ever since. EMS was called who brought the patient to the ED.	
**Past Medical History**	She is G1P1 and seven days postpartum after an uncomplicated spontaneous vaginal delivery. No prior seizures or history of epilepsy.
**Past Surgical History**	None
**Medications**	Prenatal Vitamins
**Allergies**	None
**Social History**	Lives with husband and her newborn baby girl; currently breastfeeding; denies smoking, alcohol use, or illicit drugs
**Family History**	None
**Review of Systems**	Otherwise, negative

**STIMULUS 3:** Physical Exam Findings

**Physical Examination**	
**General**	Confused, sleepy
**HEENT**	Normocephalic, atraumatic, pupils equal and reactive to light, small abrasion to the lateral tongue
**Neck**	No nuchal rigidity, midline tenderness, or step off
**Respiratory**	Normal
**Cardiac**	Normal
**Abdominal**	Normal, no tenderness or hepatosplenomegaly
**Urogenital**	Normal external genitalia, small lochia, no vaginal bleeding; Uterinefundus firm, nontender, and palpated inferior to the umbilicus
**Extremities**	2+ bilateral pedal edema, no deformity
**Neurologic**	Confused, cranial nerves grossly intact, moves all four extremities to command without apparent deficit, 3+ patellar reflexes
**Skin**	Normal

**STIMULUS 4:** ECG

**Electrocardiogram** 10
Ventricular Rate: 132 bpm
PR Interval: 110 ms
QRS Duration: 80 ms
QTc: 460 ms
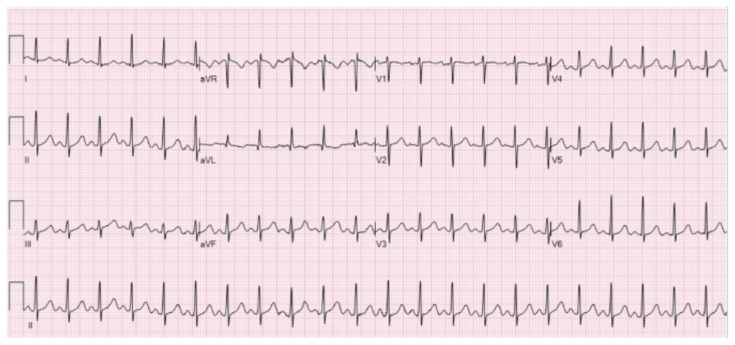

**STIMULUS 5:** Laboratory Results

**Laboratory Analysis**
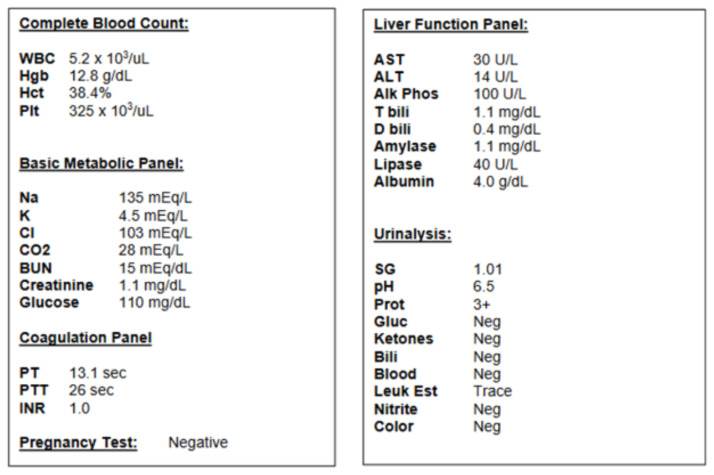

**STIMULUS 6:** Computed Tomography of the Head

**CT Head** 11
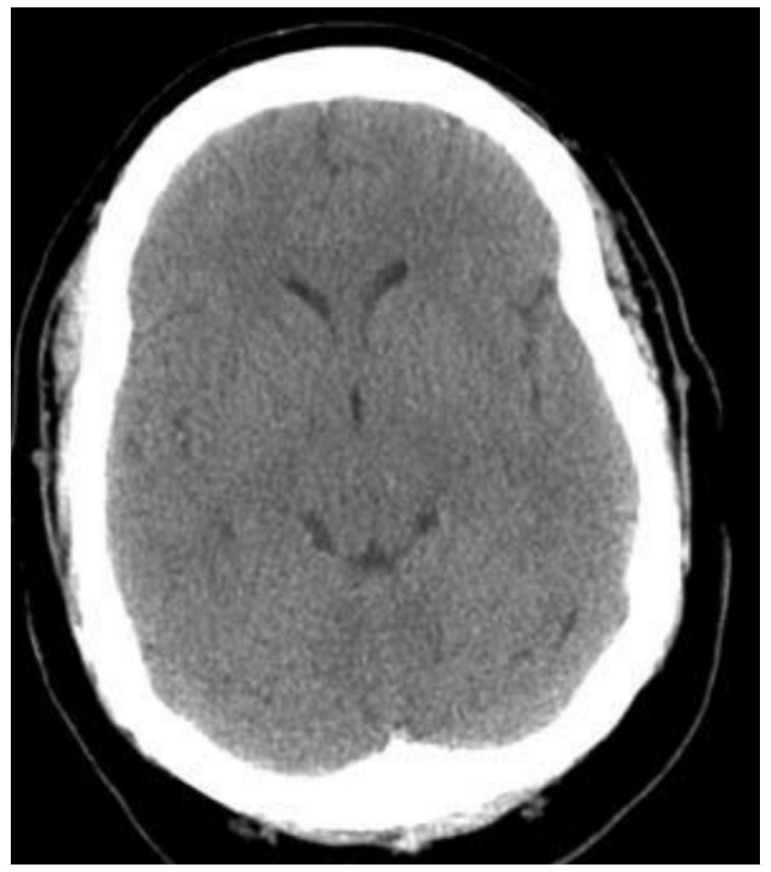

## FOR EXAMINER ONLY

### Clinical Decision-Making Case: Seizing the Diagnosis: Eclampsia Summary

**Diagnosis:** Eclampsia

**Case Summary:** A 42-year-old female presents to the ED via EMS with her husband after having seizure-like activity. The patient is very confused and unable to participate in history taking. Her husband describes an episode of loss of consciousness and generalized tonic-clonic activity. After the examinee can list questions they would ask in their HPI, it is revealed that the patient is seven days postpartum. She has been experiencing headache and blurry vision for three days. She has no previous history of seizures. She is also profoundly hypertensive.

Her lab work is notable for 3+ proteinuria with normal WBC, platelets, AST/ALT, coagulation studies, and renal function. Her ECG is sinus tachycardia. Her CT head shows no acute abnormalities.

### Clinical Decision-Making Case: Seizing the Diagnosis: Eclampsia Examiner Script

**Case Introduction:**

“Hello Doctor, this is a clinical decision-making case. There is no role playing. In response to the questions I will ask, please give me a LIST of information you would gather to come to a final diagnosis. At times, I may interrupt you to move you through the case; this is not a reflection of your performance. You will have 15 minutes to complete the case. Before we begin, do you have any questions?”

“The patient will be a 42-year-old female with seizure-like activity. Here is your task sheet for this case.” Provide the task sheet and allow the examinee to review the information provided.

Provide Learner Stimulus #1

#### HISTORY

Prompt 1:

“Here is the initial information regarding this patient. After you have read it, please give me a list of the additional historical information you would obtain from EMS or the patient’s husband.”

Prompt 2:

“You indicated you would ask the patient about X. Why is this important to you?”

**ASK THIS PROMPT TWICE ABOUT TWO SEPARATE TOPICS**

Possible examples: Why did you ask about: seizure history, recent trauma, recent illnesses, recent pregnancy, alcohol abuse or withdrawal, substance abuse history, medication taken daily, surgical history, family history of seizures, associated symptoms?

Scoring Guidelines:

Rationale: There are two points available for history taking. To receive the first point, the examinee must ask if the patient has a history of seizures. To receive the second point, the examinee must ask if the patient has a recent history of head trauma, pregnancy status, or recent illnesses (asking about any of these scenarios qualifies for the second point). These questions are critical to ask of all patients with seizures to help inform an EM physician’s diagnostic algorithm.

General Guidelines:

- If candidate begins managing the case like a standard case, examiner states, “Remember Doctor, there is no role playing in this case. Please list the additional information you want to obtain.”
- If candidate does not offer a complete list of historical information, examiner should pause long enough to allow them to list additional items, before asking “why” questions.
- If candidate mentions “past medical history,” or “social history” examiner clarifies by asking, “What specifically do you want to know about past medical/social history?”

Provide Learner Stimulus #2

“You are provided with the following additional historical information.” Show the historical information provided on Learner Stimulus #2.

#### PHYSICAL EXAMINATION

Prompt 3:

“Based on what you now know, please give me a list of specific physical examination findings you would be looking for.”

Prompt 4:

“Doctor, you examined X during the physical exam. Please explain how that would help you.”

Examples: Assessing for focal neurologic deficits, pupil irregularity, altered mental status, right upper quadrant (RUQ) tenderness or hepatomegaly, pedal/lower extremity edema, nuchal rigidity/neck stiffness, petechia/ecchymosis, evidence of trauma.

Scoring Guidelines:

Rationale: The physical exam should help inform the examinee’s differential diagnosis for the etiology of the patient’s seizures. A complete neurological exam will evaluate for mental status changes or focal neurologic deficits, increasing the likelihood of metabolic or neurovascular events. Lower extremity edema, RUQ tenderness, hepatomegaly, and signs of coagulopathy can help to assess for diagnoses on the eclampsia/HELLP spectrum. Nuchal rigidity could suggest evidence of meningitis or subarachnoid hemorrhage. Evidence of trauma will increase the likelihood of structural or traumatic intracranial abnormalities.

#### DIFFERENTIAL DIAGNOSIS

Provide Learner Stimulus #3

“You are provided with the following physical exam findings.” Show the physical exam findings provided on Learner Stimulus # 3.

Prompt 5:

“Based on what you now know, what are the top three items on your differential diagnosis based on the most likely conditions?” (If more than three conditions are mentioned, say, “Thank you for that information. Please give me your three, and only three, most likely diagnoses.”)

Appropriate differential diagnoses include:

- Eclampsia
- Subarachnoid Hemorrhage/Intracranial Hemorrhage
- Posterior Reversible Encephalopathy Syndrome (PRES)
- Hypertensive Emergency/Encephalopathy
- Thyroid Storm
- HELLP Syndrome
- Intracranial Mass
- Drug Intoxication
- Drug/ETOH Withdrawal
- Meningitis/Encephalitis
- Epilepsy
- Hypoglycemia
- Hyponatremia
- Syncope

Scoring Guidelines:

Rationale: The examinee must list eclampsia to receive the first point in this category. If their other two differential diagnoses are on the list above, they receive one point apiece. The examinee must list eclampsia to receive all three points in this category.

#### DIAGNOSTIC STUDIES

Prompt 6:

“Based on what you know and your working differential diagnosis, what, if any, diagnostic studies would you order?”

Prompt 7:

“Doctor, you ordered X. Why X?”

**ASK THIS PROMPT ONCE ABOUT A DIAGNOSTIC STUDY THAT WAS ORDERED.**

Scoring Guidelines:

Rationale: Diagnostic studies evaluating for emergent causes of seizure should be ordered. Examinees will score one point for correctly ordering pertinent laboratory analysis, including point of care (POC) glucose. These labs should be obtained to evaluate for common treatable causes of seizures, such as hypoglycemia, hypernatremia, hyponatremia, uremia, etc. Examinees will earn one point for ordering a head CT scan to evaluate for structural intracranial pathology that could cause a seizure.

Examinees will receive an additional one point for adequately explaining their rationale for ordering one of the appropriate diagnostic studies.

Prompt 8:

“The following results have been obtained. Please provide your interpretation of the following diagnostic studies.”

Please note, examiners should provide all the stimuli and results regardless of whether the examinee ordered/discussed the studies or not. While showing the examinee the associated diagnostic stimuli, examiners should ask the examinees to provide their interpretation of the diagnostic studies.

Provide Learner Stimulus #4 (Electrocardiogram - ECG)

Interpretation: ECG with sinus tachycardia, rate 132, normal axis, no ST-elevation myocardial infarction (STEMI), no evidence of serious arrhythmia.

Provide Learner Stimulus #5 (Laboratory Results)

Interpretation: Lab work is notable for 3+ proteinuria on the urinalysis with normal electrolytes, renal function, AST/ALT, CBC, and coagulation studies. If other labs were ordered by the examinee, the instructor should verbally report that all other labs are within normal limits.

Provide Learner Stimulus #6 (CT Head)

Interpretation: Normal CT head. No intracranial hemorrhage, mass, or other structural pathology.

Scoring Guidelines:

Rationale: Examinees should provide their interpretation of the pertinent laboratory studies, CT head, and ECG. Examinees will score one point for correctly interpreting the labs and identifying proteinuria on the urinalysis. Examinees will also score one point for correctly interpreting the CT head (no acute intracranial abnormality).

#### TREATMENT AND OTHER ACTIONS

Prompt 9:

“The nurse states that the patient is now having another seizure. Doctor, how are you going to manage this patient? Based on what you now know, what treatments, if any, would you order and/or what actions, if any, would you perform?”

Prompt 10:

“Doctor, you ordered X. Why X?”

**ASK THIS PROMPT TWICE ABOUT TWO SEPARATE TOPICS**

If blood pressure medication and magnesium were not both administered, please provide the following information:

“Repeat vitals are Blood Pressure: 180/95 mmHg, Heart Rate: 100, Respiratory Rate 16, Oxygen Saturation 95%. Are there any other interventions or treatments you would like to administer at this time?”

Scoring Guidelines:

Rationale: The examinee receives one point for ordering Magnesium 4–6g IV to control the patient’s eclamptic seizures. The examinee receives an additional point for ordering EITHER Labetalol 10–40mg IV, Hydralazine 10–20mg IV, OR Nicardipine IV infusion 5 mg/hr - 15 mg/hr to reduce the blood pressure. Nitroglycerin IV infusion can also be used as an adjunct but should not be the only antihypertensive medication administered. To receive points, the medication route must be intravenous, and dosing provided must be in the acceptable range. Repeat vitals can be provided to the examinee if they do not order both intravenous magnesium and anti-hypertensive medication. This prompt can help remind the examinees about the initial vitals and cue them to provide medication to treat both seizure and hypertension.

The examinee will also receive one point per correct rationale explaining the treatment choices for magnesium (one point) and for an antihypertensive medication (one point). The examiner may elicit the rationale by repeating prompt 10: “Doctor, you ordered X. Why X?” The examinee should explain the indications for administering magnesium to treat eclamptic seizures and for providing antihypertensive medication to manage elevated blood pressure in the setting of eclampsia.

#### FINAL DIAGNOSIS

Prompt 11:

“Based on everything you know about this case, what is your final diagnosis?” Correct diagnosis: Eclampsia

Scoring Guidelines:

Rationale: To receive the point for final diagnosis, the examinee must use the term eclampsia. If the candidate mentions a vaguely similar diagnosis, such as seizures, epilepsy, or post-partum seizures, the examiner should ask “Can you be more specific about the diagnosis?”

#### BROAD PRINCIPLES

Prompt 12:

“Not specific to this case, doctor, but what immediate actions should be prioritized when caring for patients who present in status epilepticus?”

Scoring Guidelines:

Rationale: Status epilepticus is a time-sensitive neurological emergency where “time is brain.” Emergency physicians should prioritize assessing and stabilizing the ABCs (Airway, Breathing, Circulation), continuous cardiac and pulse oximetry monitoring, applying supplemental oxygen, and rapidly obtaining intravenous access. In general, benzodiazepines (eg, Midazolam 10 mg IM, Lorazepam 4 mg IV, or Diazepam 10 mg IV) are considered first line medications for status epilepticus and should be administered as soon as possible.

Additionally, clinicians must prioritize glucose checks early, because hypoglycemia is a common, reversible cause of seizure activity (the “ABCDEFG” mnemonic: ABCs + Don’t Ever Forget Glucose).

The examinee should receive one point for accurately describing immediate supportive care actions or identifying appropriate benzodiazepine medications for the treatment of status epilepticus. Instructors should keep track of time and stop the examinee after they discuss one or two appropriate immediate actions to ensure adequate time remains for the rest of the case.

#### DISPOSITION

Prompt 13:

“Based on what you know, what should be the disposition of this patient?”

Prompt 14:

“Why would you [admit/discharge] this patient?”

Scoring Guidelines:

Rationale: The examinee receives a point for verbalizing that the patient should be admitted to the OB/GYN service or the ICU with OB/GYN consulted. To receive the second point in this category, the examinee should explain the reason for admission. Examples of this include continued seizure monitoring and anti-epileptic dosing, blood pressure control with IV antihypertensives, IV magnesium administration, serial neurologic exams, and monitoring of hepatic/renal function.

#### TRANSITION OF CARE

Prompt 15:

“What information would you provide to the patient and family at the time of disposition?”

Scoring Guidelines:

Rationale: To successfully receive one point for transition of care, the examinee should effectively discuss the diagnosis, treatment, and disposition with the patient and her husband in layman terms.

***Thank you, Doctor. That concludes this case.***

***Please tear up your notes.***

## CERTIFYING EXAM ASSESSMENT

*Clinical Decision-Making Case: Seizing the Diagnosis: Eclampsia*

Learner: _________________________________________

**Summative and formative comments:**

## DEBRIEFING AND EVALUATION PEARLS

### Clinical Decision-Making Case: Seizing the Diagnosis: Eclampsia

***Case Overview:*** Eclampsia in a postpartum female presenting with seizures

This case involves a postpartum female presenting with several days of headache, blurry vision, and a new-onset seizure. Upon evaluation in the ED, she is found to have elevated blood pressure, pedal edema, and proteinuria. The presumptive diagnosis of **eclampsia** should be made early, and magnesium sulfate and anti-hypertensive medications initiated promptly.

#### Initial Differential Diagnosis

In patients presenting with headache, hypertension, and new-onset seizure, the differential diagnosis encompasses several critical categories of pathology. These include structural (eg, intracranial hemorrhage, mass, or edema), infectious (eg, CNS infection), toxic-metabolic (eg, toxic ingestion, withdrawal), and vascular etiologies (eg, hypertensive emergency, PRES, eclampsia).^3^ Prompt identification of these conditions is essential due to their potential for rapid clinical deterioration.

Once the patient’s recent pregnancy history is identified, **eclampsia** should rise to the top of the differential. Eclampsia is an emergent condition on the spectrum of hypertension during pregnancy.^4^

#### Categories of Hypertension in Pregnancy^5^–^7^

1. **Chronic Hypertension:** Usually presents before pregnancy.
2. **Gestational Hypertension:** Isolated hypertension after 20 weeks of gestation without other symptoms.
3. **Preeclampsia:** Hypertension with proteinuria, with or without edema.
4. **Eclampsia:** Preeclampsia plus seizures or unexplained coma.
5. **HELLP Syndrome:** Hemolysis, Elevated Liver Enzymes, Low Platelets.

#### Important Notes on Timing

- Preeclampsia and eclampsia can occur from the 20th week of gestation up to 10 days postpartum. However, rare cases have been reported as late as 6 weeks post-delivery.^5^
- Eclampsia can develop **without prior symptoms of preeclampsia** or proteinuria.^8^

#### Management Guidelines for Eclampsia

1. **Definitive Treatment:** Delivery of the fetus is the only definitive treatment. In postpartum cases, focus on stabilizing the patient.^5^
2. **Magnesium Sulfate:**
  - **Dose:** 4–6 g IV over 10–20 minutes, followed by continuous infusion of 1–2 g/hour.^5^
  - **Properties:** Both antiepileptic and antihypertensive effects.
  - **Monitor for toxicity:** Loss of deep tendon reflexes, respiratory depression, and bradyarrhythmia. Treatment: Calcium gluconate.^5^
3. **Antihypertensive Medications:**
  1. **Options:** Hydralazine, Labetalol, or Nicardipine are safe and effective. Nitroglycerin can also be used as an adjunct medication but is not considered a first-line agent.^9^
  2. **Avoid:** ACE inhibitors, which are contraindicated in pregnancy.^5^
4. **Seizure Evaluation:**
  1. A thorough workup including brain imaging and labs is frequently indicated for new onset seizures for pregnant and non-pregnant patients.^9^
  2. For new-onset seizures before 20 weeks of gestation or postpartum, prioritize evaluation for CNS pathology as the most likely etiology.^9^
  3. After 20 weeks of gestation, eclampsia should be highest on the differential for new onset seizures.^9^
  4. Benzodiazepines and anti-epileptic medications should also be considered to treat seizures in the peripartum period. Teratogenicity should always be considered when determining the risks and benefits of treatment during pregnancy. However, initial stabilization of the mother is usually associated with improved outcomes for both the mother and fetus.^5^
5. **Admission and Consultation:**
  1. Patients with sustained blood pressure ≥140/90 mmHg and/or symptoms related to hypertension/eclampsia require **stat obstetrics consultation** and hospital admission.^5^
  2. Intensive care unit admission may be required for refractory seizures, unstable vitals, or lifesaving interventions such as intubation.^5^
